# Epigenetics at the crossroads of secondary growth regulation

**DOI:** 10.3389/fpls.2022.970342

**Published:** 2022-08-05

**Authors:** Vera Inácio, Raquel Santos, Rafael Prazeres, José Graça, Célia M. Miguel, Leonor Morais-Cecílio

**Affiliations:** ^1^BioISI – Instituto de Biosistemas e Ciências Integrativas, Faculdade de Ciências, Universidade de Lisboa, Lisbon, Portugal; ^2^Forest Research Centre (CEF), Institute of Agronomy, Universidade de Lisboa, Lisbon, Portugal; ^3^Linking Landscape, Environment, Agriculture and Food (LEAF), Institute of Agronomy, Associated Laboratory TERRA, Universidade de Lisboa, Lisbon, Portugal

**Keywords:** secondary meristem, phellogen, cork, vascular cambium, DNA methylation, histone posttranslational modifications

## Abstract

The development of plant tissues and organs during post-embryonic growth occurs through the activity of both primary and secondary meristems. While primary meristems (root and shoot apical meristems) promote axial plant growth, secondary meristems (vascular and cork cambium or phellogen) promote radial thickening and plant axes strengthening. The vascular cambium forms the secondary xylem and phloem, whereas the cork cambium gives rise to the periderm that envelops stems and roots. Periderm takes on an increasingly important role in plant survival under climate change scenarios, but it is also a forest product with unique features, constituting the basis of a sustainable and profitable cork industry. There is established evidence that epigenetic mechanisms involving histone post-translational modifications, DNA methylation, and small RNAs play important roles in the activity of primary meristem cells, their maintenance, and differentiation of progeny cells. Here, we review the current knowledge on the epigenetic regulation of secondary meristems, particularly focusing on the phellogen activity. We also discuss the possible involvement of DNA methylation in the regulation of periderm contrasting phenotypes, given the potential impact of translating this knowledge into innovative breeding programs.

## Introduction

Meristems allow plants to grow throughout their lifespan by continuously or seasonally producing cells that differentiate into specialized tissues. Apical meristems like the shoot apical and root apical meristems are responsible for primary growth, i.e., axial plant extension, while secondary or lateral meristems enable secondary growth, i.e., increase in radial thickness and strengthening plant axis. Secondary meristems envelop stems and roots of woody and some herbaceous species and include the vascular cambium and the cork cambium or phellogen (Turley and Etchells, 2022).

The vascular cambium derivatives develop into secondary xylem toward the inside and phloem outward, essential tissues for water and nutrient transport and mechanical support (Wang et al., 2021). The phellogen derivatives develop into mostly suberized phellem or cork cells outward and phelloderm cells inward, together making the three-layered periderm that confers protection against environmental stresses (Evert, 2006).

DNA methylation, histone posttranslational modifications (hPTMs), and small RNA (sRNA) directed epigenetic modifications are implicated in plant developmental processes such as maintenance and/or repression of meristem activity and its progeny cells fate, leaf morphogenesis, floral organ formation, flowering time determination, and tissue regeneration after injury, among others (reviewed in Ikeuchi et al., 2015b; Dong et al., 2022). The interplay among several epigenetic marks affects the chromatin structure either by creating compact or loose states associated either with gene silencing or activation, respectively. DNA methylation is usually found in transcriptionally inactive chromatin, while hPTMs are related either with repressed or active chromatin domains, depending on the modification type. Histone acetylation almost invariably correlates with transcriptional activation, and histone methylation can be either associated with gene activation or silencing, according to its histone residue context (reviewed in Gallusci et al., 2017). Within the sRNAs, the short-interfering RNAs (siRNAs) have been implicated in RNA-directed DNA methylation, but mainly in transposons and repetitive DNA (Zhang et al., 2018). The micro RNAs (miRNAs) class of sRNAs have also been shown to play a role in DNA methylation (Jia et al., 2011), although their main and best characterized mode of action occurs at the post-transcriptional level by negatively regulating their target genes, which may include transcripts of genes directly involved in epigenetic modifications such as *CMT3* and *DRM2* (Duarte et al., 2013; Jha and Shankar, 2014). Additionally, siRNAs promoting DNA methylation can also be produced from miRNA loci (Chellappan et al., 2010).

Even though epigenetic mechanisms and phytohormones are key players in primary growth (reviewed in Maury et al., 2019), the knowledge of their specific roles in secondary growth is poorly understood despite the importance of secondary growth-derived biomass in ecosystems, society, and industry. Here, we provide an overview of recent findings on how epigenetic mechanisms affect plant secondary growth, particularly focusing on periderm formation, and discuss future perspectives of periderm epigenetics research.

## Epigenetic marks in vascular cambium regulation

**Figure 1:**
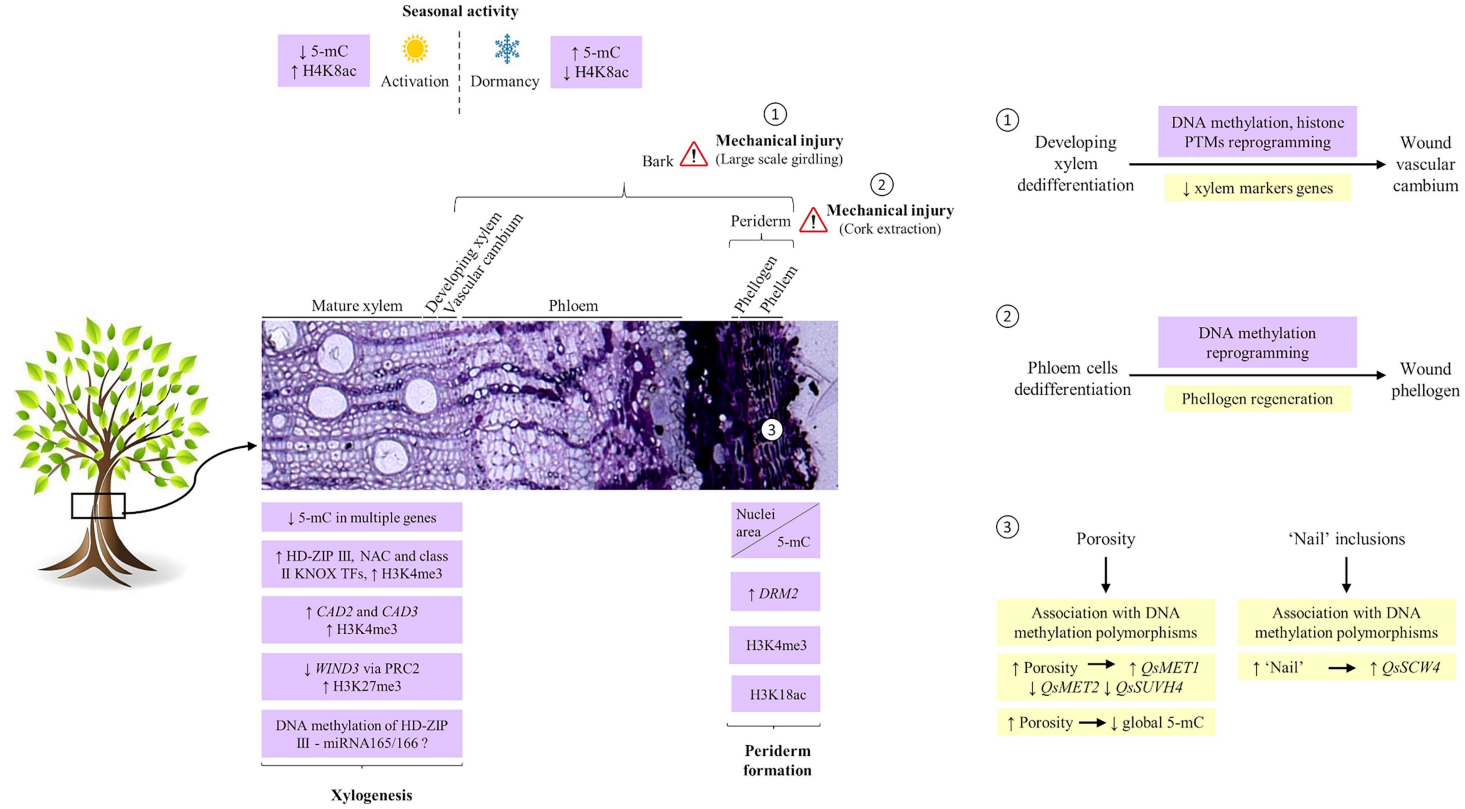
Epigenetic regulation of secondary growth. Transcription factors and genes involved in the different steps of xylogenesis are regulated, at least in part, by histone modification and DNA methylation. During the dormancy period, xylem and phloem cells show high levels of repressive marks (5-mC) and low levels of active marks (H3K8ac) while showing the opposite pattern in the active period. The dormant-active transition cycles imply DNA hypomethylation in the promoter regions of genes involved in plant hormone signalling, cell wall biosynthesis, and transcriptional regulation. In periderm, differentiation of cork cells is accompanied by drastic chromatin remodeling, evidenced by chromatin condensation and accumulation at the nuclear periphery and nuclei area decrease. Alongside, progressive increase in DNA methylation, upregulation of *QsDRM2*, and rather constant levels of H3K4me3 and H3K18ac gene activation associated marks are present. **(1)** After bark girdling, the regeneration of a wound cambium by the dedifferentiation of developing xylem cells involves DNA methylation, and histone PTMs reprogramming. **(2)** Similarly, after cork extraction, the regeneration of the phellogen is determined by a switch of phloem cells fate characterized by a reprogramming in DNA methylation patterns. **(3)** Usually under stomata, the cork cambium redifferentiates into lenticular phellogen which forms lenticular cells responsible for cork porosity. These cells are characterized by low levels of DNA methylation and are likely epigenetically regulated via DNA methylation. The ‘nail’ which is formed after randomly localized phellogen death is also potentially regulated by DNA methylation. HD-ZIP, HOMEODOMAIN-LEUCINE ZIPPER; SND, SECONDARY WALL-ASSOCIATED NAC DOMAIN; KNOX, KNOTTED-LIKE HOMEOBOX; 5-mC, 5-methylcytosine; DRM, DOMAINS REARRANGED METHYLTRANSFERASE; MET, methyltransferase; SUVH4, KRYPTONYTE histone methyltransferase; SWC4, SWR1-complex protein 4; PTMs, posttranslational modifications; H4K8ac, acetylation of histone H4 at lysine 8; H3K4me3, trimethylation of histone H3 at lysine 4; H3K18ac, acetylation of histone H3 at lysine 18; *Qs, Quercus suber*.

Epigenetic regulation of vascular cambium activity has been identified during dormancy and activation periods (Schrader et al., 2004; Conde et al., 2013; Chen et al., 2021), xylogenesis (Du et al., 2011; Robischon et al., 2011; Zhao et al., 2015; Hussey et al., 2017; Hou et al., 2020), and in the secondary vascular tissue regeneration after injury (SVTR; Zhang et al., 2011; Lee and Seo, 2018; Figure 1).

### Xylogenesis

Secondary xylem differentiation (xylogenesis) follows four steps: (i) cambium cell division with inward daughter cells enlargement and shape modification, (ii) secondary cell wall formation with deposition of cellulose, hemicellulose, and lignin, and (iii) developmentally induced programmed cell death (dPCD) whereby mature xylem cells turn into tracheary elements (Rathgeber et al., 2016). Over time, different molecular models of wood formation from the vascular cambium were proposed and have been extensively reviewed (Eckes-Shephard et al., 2022), but its epigenetic regulation is yet to be explored.

Recently, active DNA demethylation in CH, CHG, and CHH contexts in multiple genes has been identified in *Arabidopsis* as a key player in vascular development, namely in the tracheary elements differentiation step (Wei et al., 2022).

In developing xylem of *Eucalyptus grandis*, homologs of the HOMEODOMAIN-LEUCINE ZIPPER (HD-ZIP) III transcription factors *ARABIDOPSIS THALIANA* HOMEOBOX 8 (ATHB8), ATHB15, and REVOLUTA (REV), of SECONDARY WALL-ASSOCIATED NAC DOMAIN 1 (SND1) and SND3, and class II KNOX KNOTTED-LIKE HOMEOBOX OF *ARABIDOPSIS THALIANA* 7 (KNAT7) that regulate xylem development, as well as cambial markers (homologs of Arabidopsis SHOOT MERISTEMLESS – STM, KNAT1, RESPONSE REGULATOR 12 – ARR12, OBF BINDING PROTEIN 1 – OBP1, and WUSCHEL RELATED HOMEOBOX 4 – WOX4) were enriched with H3K4me3, a hallmark of transcription, while phloem markers (homologs of Arabidopsis ALTERED PHLOEM DEVELOPMENT – APL, CLAVATA3/ESR-RELATED 41 – CLE41, CLE44, and KANADI) had a greater enrichment with H3K27me3, associated with repressed genes (Hussey et al., 2017). In turn, HD-ZIP III like REV, PHABULOSA (PHB), PHAVOLUTA (PHV), ATHB15, and ATHB8 which have different functions in xylem differentiation, cambial activity, and lignification are controlled by miRNA165/166 (Ramachandran et al., 2017). These miRNAs seem to mediate DNA methylation of their target genes (Bao et al., 2004), but the mechanism involved is still unclear (Teotia et al., 2017).

In *E. grandis*, lignin biosynthesis core set genes, like *CINNAMYL ALCOHOL DEHYDROGENASE* 2 and 3 (*CAD2* and *CAD3*), involved in the last formation steps of different lignin types (lignin S, G, or H; Carocha et al., 2015) were enriched with the H3K4me3 activation mark, contributing to their upregulation and lignin deposition (Hussey et al., 2017).

### Seasonal activity

The activity of cambial cells also implies a seasonal pattern of development. In *Populus tomentosa*, establishment of the dormant state in the cambial meristem was associated with the reduction of the transcriptome complexity by inhibition of genes that enhance cambial cell divisions (Schrader et al., 2004). Recently, it was described that the transition from dormancy to the active stage involves not only transcription but also epigenetic changes in the cambium cells so that wood production can take place (Chen et al., 2021). Even though the mechanisms are not entirely known, the expression of genes involved in plant hormone signal transduction, cell wall biosynthesis, and transcriptional regulation during the dormant-active transition stage is potentially regulated by DNA hypomethylation of their promoter regions (Chen et al., 2021).

In poplar stems, the DNA methylation levels measured by immunodetection of 5-methylcytosine in phloem and xylem cells were significantly higher during the dormancy period than in the active period, while the levels of acetylation of histone H4 at lysine 8 (H4K8ac) showed the opposite pattern (Conde et al., 2013). This study established that epigenetic regulation plays a part in the differential gene expression during the growth and arrest periods in xylem and phloem tissues.

### Secondary vascular tissue regeneration after injury (SVTR)

The vascular cambium also forms the secondary phloem outward that, along with the periderm, comprise the bark. After large-scale girdling of trees, bark removal peels off the vascular cambium and regeneration occurs from differentiating xylem cells to form a new cambium (Chen et al., 2013). This SVTR involves mainly three stages: (i) callus formation and xylem cell dedifferentiation, (ii) emergence of sieve elements, and (iii) wound cambium formation (Chen et al., 2013). The wound xylem formation implies multiple transcriptome changes that have been extensively studied in pine tree (Chano et al., 2017). In early-SVTR of poplar, genes like DNA and histone methyltransferases and chromatin remodelers displayed significant expression changes during xylem dedifferentiation along with downregulation of xylem marker genes and xylem-specific transcription factors (Zhang et al., 2011). These results suggest that during pre-callus formation, DNA methylation and chromatin remodeling underlie xylem cell fate switch and regeneration of secondary vascular tissue (Ojolo et al., 2018).

These results agree with the known role of hPTMs and DNA methylation in cellular plasticity and reprogramming necessary for dedifferentiation and regeneration (reviewed in Ikeuchi et al., 2019). Indeed, during normal development, there are many reprogramming regulators epigenetically silenced that are activated by wounding and/or hormonal signaling (reviewed in Ikeuchi et al., 2019). For instance, the DNA methyltransferase MET1 prevents the precocious expression of WUSCHEL (WUS) transcription factor, responsible for *de novo* establishment of shoot stem cell niche, downstream of cytokinin signaling during callus induction (Liu et al., 2018). During shoot regeneration, WUS is activated (Ikeda et al., 2009) after removal of the repressive histone mark H3K27me3 and downstream binding of ARABIDOPSIS RESPONSE REGULATORs (ARRs) ARR1, ARR2, ARR10, and ARR12 transcriptional activators to PHB, PHV, and REV HD-ZIP III transcription factors (Zhang et al., 2017).

During the wound-induced-callus formation in Arabidopsis, the *WOUNDINDUCED DEDIFFERENTIATION 1–4 (WIND1-4)* genes are activated and promote cytokinin responses to allow callus formation (Iwase et al., 2011). *WIND1* and *WIND3* are repressed by the POLYCOMB REPRESSIVE COMPLEX 2 (PRC2) through the deposition of H3K27me3 (Holec and Berger, 2012) to maintain differentiated states of somatic cells of Arabidopsis root (Ikeuchi et al., 2015a). Besides wound-induced-callus formation, *WIND* transcription factors also promote tracheary element formation and vascular reconnection by transcriptionally activating genes involved in cellular reprogramming, vascular formation, and defense response (Iwase et al., 2021). WIND1 targets showed H3K27me3 repressive marks before wounding and H3K9/14 ac and H3K27ac after wounding (Rymen et al., 2019).

## Epigenetic evidence in periderm development

The periderm formation involves several processes: phellogen initiation and cell proliferation, radial cell elongation, secondary cell wall formation through the deposition of suberin and waxes, and dPCD (Inácio et al., 2021; Serra et al., 2022). Despite the enormous progress in understanding the molecular mechanisms underlying vascular cambium regulation, very few regulators or potential regulators of phellogen activity and phellem differentiation have been identified (Miguel et al., 2016; Verdaguer et al., 2016; Xiao et al., 2020; Ye et al., 2021). These include auxin and cytokinin signaling and downstream action of transcription factors like WOX4, BREVIPEDICELLUS (BP)/KNOTTED-LIKE FROM *ARABIDOPSIS THALIANA* 1 (KNAT1), LATERAL ORGAN BOUNDARIES DOMAIN 3 (LBD3) and LBD4, and SHORT-ROOT-like gene *PtSHR2B* from *Populus* (reviewed in Serra et al., 2022). As seen during vascular cambium activity, new evidence suggests that phellogen activity might be under epigenetic control (Figure 1; Ramos et al., 2013; Inácio et al., 2017, 2018), as reviewed in the following sub-sections.

### Chromatin marks during periderm formation

Developmentally induced programmed cell death involved in cork cells differentiation is accompanied by chromatin condensation and reallocation to the nuclear periphery, nuclei area decrease, and progressive increase in *de novo* DNA methylation (Inácio et al., 2018). It is reasonable to assume that the chromatin condensation during cork cells differentiation is the result of such an increase in *de novo* DNA methylation. Accordingly, the *de novo* methyltransferase cork oak *QsDRM2* was highly expressed during cork formation (Ramos et al., 2013; Inácio et al., 2018).

In potato tuber periderm, the interactome of VASCULAR TISSUE SIZE (VAS), an inducer of procambial activity (Van der Graaff et al., 2002) related to phellogen initiation (Vulavala et al., 2019), included a shoot-apical meristem cell-cycle regulator, and cell-wall synthesis and chromatin regulation proteins (Vulavala et al., 2019). Additionally, the interactome of a phellogen inactivation-related PHDZnP/Homeobox protein (HAT3; Vulavala et al., 2019), which controls meristem activity in established meristems (Turchi et al., 2013), involved regulators of brassinosteroid-responsive genes *via* histone modification, mostly cell-wall elongation genes important for proper development and differentiation (Vulavala et al., 2019).

While DNA methylation is highly represented in older living cork cells, the newly formed ones showed a high level of gene activation associated marks (Inácio et al., 2018). In fact, an increase in H3K4me3 levels from the phellogen to differentiating cork cells while similar levels of H3K18ac in all periderm cells were observed. Genes involved in secondary cell wall deposition, as well as PCD-associated genes, might be upregulated in cork cells through H3K4me3 modification. This would be consistent with previous findings in senescent Arabidopsis leaves and developing xylem showing a strong correlation between the upregulation of senescent-associated and secondary cell wall genes and H3K4me3 enrichment (Brusslan et al., 2015; Hussey et al., 2017).

Phellem differentiation is also potentially regulated by miRNAs since 13 miRNAs belonging to seven families already described as involved in vascular development were identified in phellem tissue (Chaves et al., 2014). Two of them, Qsu-miR-2P and Qsu-miR-7P, target a histone deacetylase and a histone acetyltransferase, indicating that these miRNAs may play a role in regulating histone acetylation involved, in turn, in cork differentiation.

### Epigenetic reprogramming during phellogen regeneration

When the phellogen dies either by a mechanical injury or by unknown factors, a new phellogen is formed, rapidly producing a traumatic or wound periderm, ensuring the viability of the tree (Pereira, 2007). It is this regeneration capacity that allows the exploitation of the cork oak tree (*Quercus suber* L.) in a sustained way and throughout the tree’s lifespan, by successive cork extractions. Due to its highly active and long-living phellogen, cork oak produces an exceptionally thick periderm or cork, used in several industrial applications (Pereira, 2007). The cork harvesting is performed during the more intense period of phellogen activity taking advantage of the fragility of the phellogen cell walls and the more recently divided cork cells, which are easily torn apart allowing the removal of cork without affecting the survival of the trees (Pereira, 2007). When the cork is removed, the phellogen is destroyed exposing the underlying secondary phloem. However, as a response to the trauma, a new cambium is rapidly regenerated by a process of meristematic activation in the outer, non-conducting, phloem cells, forming the new traumatic phellogen (Pereira, 2007). In cork oak, the original phellogen and contiguous differentiating cork cells showed distinct patterns of DNA methylation when compared with the traumatic phellogen (Inácio et al., 2017). This leads to the suggestion that the somatic-to-meristematic transition is probably epigenetically modulated *via* DNA methylation as hypothesized for secondary vascular tissue regeneration after girdling (Zhang et al., 2011).

### Epigenetic modulation of periderm phenotypic variability

The periderm is mostly composed of cork cells but is variable in its cellular characteristics. The cork tissue is interrupted by discontinuities such as the lenticels or pores (cork porosity) and the inclusion of heavily lignified phloem cells (“nail”) which are dependent on the phellogen activity, and largely contribute to periderm variability. In *Q. suber* commercial cork, these are very relevant aspects since “cork quality,” which dramatically affects its technical performance and economic value, is conditioned by the lenticels pattern and eventual “nail.” Lenticels are produced by the lenticular phellogen usually under stomata to permit gaseous exchange (Evert, 2006). This phellogen is highly active producing several filling cells that disaggregate to form the pores. Inácio et al. (2018) observed very low levels of DNA methylation in the cork oak lenticular phellogen corroborating its intense meristematic activity. The lenticel filling cells also showed faint DNA methylation (Inácio et al., 2018), supporting the lower global DNA methylation levels found in periderms with high porosity (Ramos et al., 2013).

Although no reports exist on the genetic control of periderm phenotypic variability, evidence in woody species suggests that such control underlies naturally occurring bark phenotypic variation (Bdeir et al., 2017). In addition to genetic, epigenetic variation is subject to selection and can contribute to rapid adaptive responses in long-lived trees (Sow et al., 2018), particularly in wood formation. Epigenetic quantitative trait *loci* were identified for wood property traits in both full-sib progeny and natural *Populus* populations (Lu et al., 2020). In the last decade, indications that periderm phenotypic variability may also be under epigenetic control *via* DNA methylation were disclosed (Ramos et al., 2013; Inácio et al., 2017, 2018). DNA methylation polymorphisms were found statistically associated with lenticel characters across three populations in different edaphoclimatic conditions (Inácio et al., 2017). Although the genomic location of these polymorphisms is unknown, the study suggested that DNA methylation is potentially involved in the lenticular phellogen activity. Accordingly, the gene expression of DNA methyltransferases *QsMET1* and *QsMET2* and histone methyltransferase *QsSUVH4* was correlated with lenticel characters (Inácio et al., 2018). Moreover, putative stomatal/lenticular-associated genes, known to be controlled through DNA methylation (Yamamuro et al., 2014), showed differential expression in periderms with contrasting cork porosity (Teixeira et al., 2014).

When, apparently at random, small paths of the phellogen die and a new one is formed within the non-conducting phloem, isolating phloem sclerified cells inside the suberized cork tissue, the “nail” is produced (Pereira, 2007). No factors controlling this randomly localized death have been identified so far; nevertheless, it has been hypothesized that DNA methylation could also have a role in this process in cork oak (Inácio et al., 2017) since a DNA methylation polymorphism was found statistically associated with a low percentage of “nail” (Inácio et al., 2017). Moreover, regulation by SWR1C remodeling complexes and/or NuA4 histone acetyltransferase complex could also be involved since *QsSWC4*, which codes for SWR1-complex protein 4 involved in transcriptional regulation (Bieluszewski et al., 2015) by targeting gene promoters in Arabidopsis (Mozgová et al., 2015), was upregulated and its expression was positively significantly correlated with periderms with a high level of ‘nail’ (Ramos et al., 2013).

## Future perspectives

Very few epigenetic players were functionally characterized during secondary meristem activity, and the regulatory network involved is far from being understood. Thus, uncovering the mechanisms behind the activity of epigenetic regulators during secondary growth will be an important goal for future studies.

In periderm research, how or whether DNA methylation, hPTMs, and/or associated miRNAs contribute to the determination of phellogen derivative cells’ fate (i.e., either differentiate as cork cells or lenticular cells) are still open questions. Lenticular channels with different characteristics (i.e., dimension, shape, number per unit area, and distribution) result from a distinct lenticular phellogen activity throughout the years that, together with the randomly localized-phellogen death, contribute to periderm phenotypic variability. How exactly epigenetic mechanisms modulate this differential activity or random death of the phellogen is another issue to be investigated. Determining to which extent epigenetic variation accounts for the phenotypic variation in species with high genetic variability is extremely challenging. In fact, the interaction between genetics, epigenetics, and the environment should be considered.

To establish a causal link between epigenetic marks and the activity of secondary meristems, functional studies are essential. Multi-omics data integration, including methylome, genome, transcriptome, and proteome at a phellogen-cell level in wild-type and epigenetic mutants in periderm model species will be essential for the identification of epigenetically regulated-candidate genes.

## Author contributions

VI suggested and designed the mini-review article. VI, RP, and RS worked on the original draft of the manuscript. VI, JG, CM, and LM-C revised and finalized the article. RS and VI designed Figure 1 and LM-C finalized. All authors contributed to the article and approved the submitted version.

## Funding

This work was funded by Portuguese Fundação para a Ciência e a Tecnologia (FCT) I.P. through national funds within the scope of project PTDC/ASP-SIL/1717/2020. The authors also acknowledge the Centre Grants UIDB/04046/2020 and UIDP/04046/2020 (BioISI), UID/AGR/04129/2020 (LEAF), and UID/AGR/00239/2019 (CEF), all funded by FCT, for the provided support. RS is the recipient of a fellowship from BioSys2 PhD programme 298 PD65-2012 (UI/BD/153490/2022) from FCT.

## Conflict of interest

The authors declare that the research was conducted in the absence of any commercial or financial relationships that could be construed as a potential conflict of interest.

## Publisher’s note

All claims expressed in this article are solely those of the authors and do not necessarily represent those of their affiliated organizations, or those of the publisher, the editors and the reviewers. Any product that may be evaluated in this article, or claim that may be made by its manufacturer, is not guaranteed or endorsed by the publisher.
